# Identification and validation of a platelet-related signature for predicting survival and drug sensitivity in multiple myeloma

**DOI:** 10.3389/fphar.2024.1377370

**Published:** 2024-05-16

**Authors:** Zhili Lin, Quanqiang Wang, Ziwei Zheng, Bingxin Zhang, Shujuan Zhou, Dong Zheng, Zixing Chen, Sisi Zheng, Shuxia Zhu, Xinyi Zhang, Enqing Lan, Yu Zhang, Xuanru Lin, Qiang Zhuang, Honglan Qian, Xudong Hu, Yan Zhuang, Zhouxiang Jin, Songfu Jiang, Yongyong Ma

**Affiliations:** ^1^ Department of Hematology, The First Affiliated Hospital of Wenzhou Medical University, Wenzhou, China; ^2^ Department of Hepatobiliary Surgery, The Second Affiliated Hospital and Yuying Children’s Hospital of Wenzhou Medical University, Wenzhou, China; ^3^ Key Laboratory of Intelligent Treatment and Life Support for Critical Diseases of Zhejiang Province, Wenzhou, China; ^4^ Zhejiang Engineering Research Center for Hospital Emergency and Process Digitization, Wenzhou, China

**Keywords:** multiple myeloma, platelet, prognostic gene signature, immune microenvironments, biological functions, sensitivity to chemotherapeutic agents

## Abstract

**Background:** Significant progress has been achieved in the management of multiple myeloma (MM) by implementing high-dose therapy and stem cell transplantation. Moreover, the prognosis of patients has been enhanced due to the introduction of novel immunomodulatory drugs and the emergence of new targeted therapies. However, predicting the survival rates of patients with multiple myeloma is still tricky. According to recent researches, platelets have a significant impact in affecting the biological activity of tumors and are essential parts of the tumor microenvironment. Nonetheless, it is still unclear how platelet-related genes (PRGs) connect to the prognosis of multiple myeloma.

**Methods:** We analyzed the expression of platelet-related genes and their prognostic value in multiple myeloma patients in this study. We also created a nomogram combining clinical metrics. Furthermore, we investigated disparities in the biological characteristics, immunological microenvironment, and reaction to immunotherapy, along with analyzing the drug susceptibility within diverse risk groups.

**Results:** By using the platelet-related risk model, we were able to predict patients’ prognosis more accurately. Subjects in the high-risk cohort exhibited inferior survival outcomes, both in the training and validation datasets, as compared to those in the low-risk cohort (*p* < 0.05). Moreover, there were differences in the immunological microenvironments, biological processes, clinical features, and chemotherapeutic drug sensitivity between the groups at high and low risk. Using multivariable Cox regression analyses, platelet-related risk score was shown to be an independent prognostic influence in MM (*p* < 0.001, hazard ratio (HR) = 2.001%, 95% confidence interval (CI): 1.467–2.730). Furthermore, the capacity to predict survival was further improved when a combined nomogram was utilized. In training cohort, this outperformed the predictive value of International staging system (ISS) alone from a 5-years area under curve (AUC) = 0.668 (95% CI: 0.611–0.725) to an AUC = 0.721 (95% CI: 0.665–0.778).

**Conclusion:** Our study revealed the potential benefits of PRGs in terms of survival prognosis of MM patients. Furthermore, we verified its potential as a drug target for MM patients. These findings open up novel possibilities for prognostic evaluation and treatment choices for MM.

## 1 Introduction

A cancerous plasma cell generated from bone marrow is called multiple myeloma. It is a clonal plasma cell disease that creates an excess of monoclonal immunoglobulin (Joshua, 2005). And it accounts for about 10% of all hematologic malignancies (Joshua et al., 2019). In recent times, substantial progress has been witnessed in the management of multiple myeloma, encompassing intensive therapy, transplantation of stem cells, the emergence of innovative medications, drugs for specific targets, and new immunomodulatory drugs (Joshua et al., 2019), improving survival rates for patients of all ages (Libby et al., 2014; Blimark et al., 2018). However, the clinical illness course is highly variable due to underlying molecular variance (Sonneveld et al., 2016). Although some patients achieve long periods of remission after treatment, the most patients will experience multiple relapses. Eventually, the remissions become shorter in duration, and the patients die from treatment-related complications or the disease itself (van de Donk et al., 2021). As a result, additional robust prognostic markers are required to improve forecast accuracy and to supplement classic ISS or Revised International Staging System (R-ISS) stages. A more effective risk categorization approach is also required to help with the management of MM patients.

Several studies demonstrated that platelet counts fluctuated frequently during cancer progression, indicating poor prognosis, especially in some malignant solid tumors such as colon, stomach, ovarian and lung cancers (Sierko and Wojtukiewicz, 2004; Gay and Felding-Habermann, 2011). In addition to being crucial for all stages of platelet generation and proliferation, a number of cytokines, including thrombopoietin (TPO), interleukin-6 (IL-6), interleukin-11 (IL-11), and interleukin-1b (IL-1b), are also involved in the pathology of myeloma (Anderson et al., 1999; Lauta, 2001). sP-selectin, IL-6, and TPO concentrations were observed to be significantly higher in newly diagnosed MM patients than in healthy individuals. Increased myeloma cell infiltration, platelet activation, and elevated platelet-derived growth factor (PDGF) expression in bone marrow stromal cells could all be the cause of this (Lemancewicz et al., 2014). Numerous substances that might affect the cancer microenvironment, including fibroblast growth factor (FGF) and vascular endothelial growth factor (VEGF), can be released by activated platelets. These molecules can also drive tumor angiogenesis. Additionally, platelets contribute significantly to the supply of transforming growth factor (TGF)-β, which helps tumor cells evade the immune system’s detection and destruction (Neuzillet et al., 2015; Abdol Razak et al., 2017). In addition to this, activated platelets can help bloodstream tumor cells stick to the vessel wall, evade immune evasion, and continue to exist and grow in the intended organs (Lei et al., 2022). There are growing evidences that tumor invasion and metastasis can be considerably decreased by blocking platelet activity (Xiulan et al., 2022). Experimental data indicated that platelet counts were halved and tumor growth was significantly reduced in tumor-bearing mice following administration of the anti-platelet antibody (Stone et al., 2012). Nevertheless, it is yet to be determined if these platelet-related genes are linked to the prognosis of patients with MM.

**FIGURE 1:**
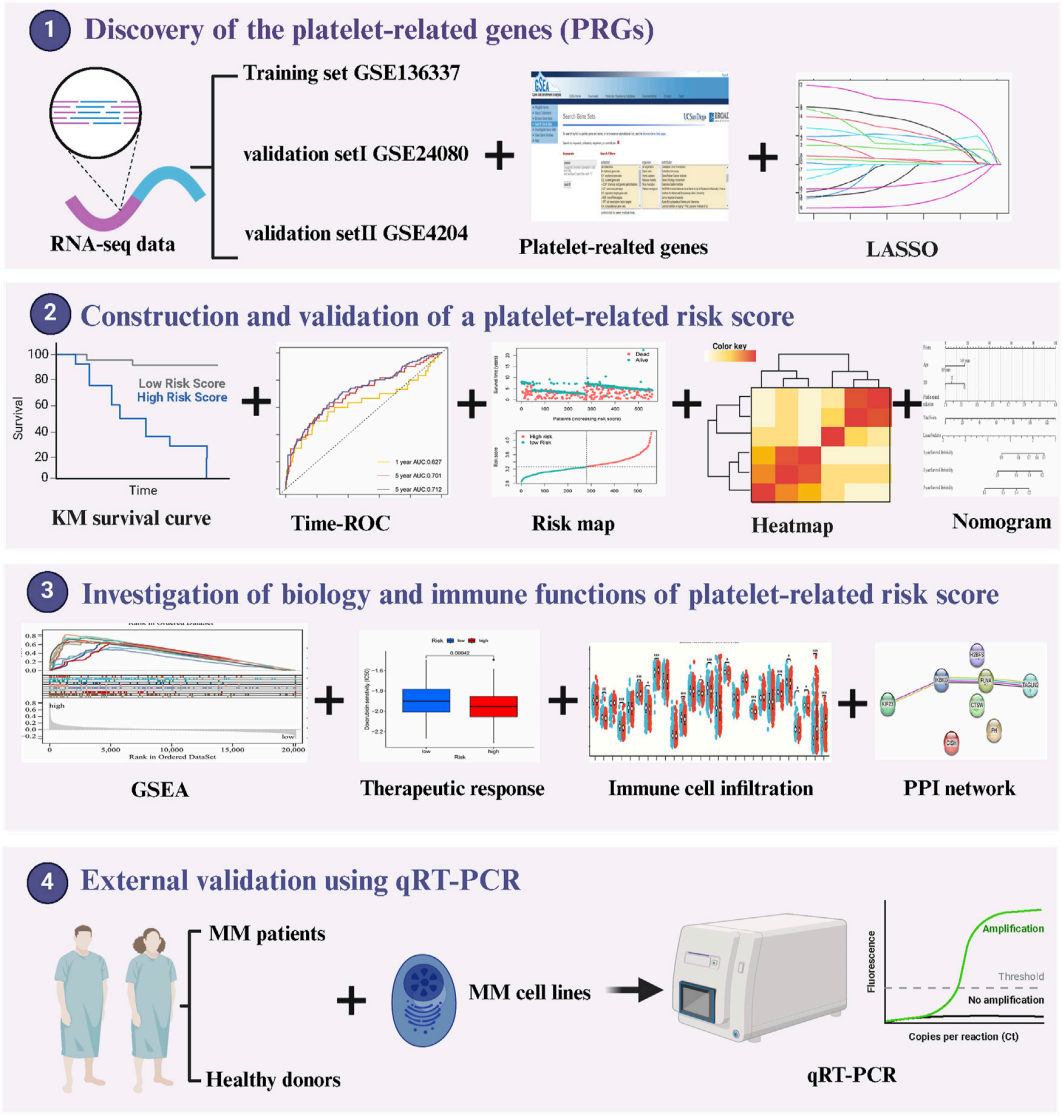
Workflow of data analysis in our study.

Consequently, creating a platelet-related predictive model to direct personalized treatment for multiple myeloma is therapeutically useful. We created a predictive model using a publicly available dataset of platelet-related genes. This study further integrated bioinformatics analysis with extensive validation using a considerable amount of samples from MM patients in order to confirm the correlation between PRGs and the prognosis of multiple myeloma. We also looked into the model’s sensitivity to immunotherapy and chemotherapy drugs. The prognostic accuracy of ISS and R-ISS was greatly improved with the addition of our platelet-related model. Figure 1 summarized the procedure for data analysis. In conclusion, this study not only provides a new factor that predicts the prognosis of multiple myeloma, but also provides new directions for multiple myeloma in targeted therapy and immunotherapy research.

## 2 Materials and methods

### 2.1 MM data collection

We used the Gene Expression Omnibus (GEO) database (https://www.gsea-msigdb.org/gsea/index.jsp/) to collect gene expression profiles and relevant clinical data, and this study employed three different datasets, namely, GSE136337, GSE4204, and GSE24080. In preparation for subsequent analysis, the expression profiles underwent a log2 transformation. For a comprehensive overview of the clinicopathological and survival data, please refer to Table 1 **.** In this study, platelet-related genes were extracted from the platelet-related genomes available in the Gene Set Enrichment Analysis (GSEA) database (http://www.gsea-msigdb.org/gsea/msigdb). A grand total of 1247 genes were chosen for subsequent investigation. Supplementary Table S1 contains information about the genes that overlap.

**TABLE 1:** Clinical co-variates of the training and validation cohorts.

Characteristics	Training cohort GSE136337 (N = 415)	Validation cohort GSE24080 (N = 559)	Validation cohort GSE4204 (N = 537)
Sex			
Female	158 (38.1%)	222 (39.7%)	-
Male	257 (61.9%)	337 (60.3%)	-
Age			
<65 years	299 (72.0%)	433 (77.5%)	-
≥65 years	116 (28.0%)	126 (22.5%)	-
Albumin			
≥3.5 g/dL	331 (79.8%)	482 (86.2%)	-
<3.5 g/dL	84 (20.2%)	77 (13.8%)	-
β2M			
<3.5 mg/L	197 (47.5%)	320 (57.2%)	-
3.5–5.5 mg/L	106 (25.5%)	125 (22.4%)	-
≥5.5 mg/L	112 (27.0%)	114 (20.4%)	-
LDH			
≤250 U/L	392 (94.5%)	509 (91.1%)	-
>250 U/L	23 (5.5%)	50 (8.9%)	-
Del (17p)			
False	400 (96.4%)	-	-
True	15 (3.6%)	-	-
t (4,14)			
FALSE	401 (96.6%)	-	-
True	14 (3.4%)	-	-
t (14,16)			
FALSE	414 (99.8%)	-	-
True	1 (0.2%)	-	-
ISS			
I	163 (39.3%)	296 (53.0%)	-
II	133 (32.0%)	149 (26.7%)	-
III	119 (28.7%)	114 (20.3%)	-
R-ISS			
I	149 (35.9%)	-	-
II	243 (58.6%)	-	-
III	23 (5.5%)	-	-
Risk score			
High	206 (49.6%)	279 (49.9%)	268 (49.9%)
Low	209 (50.4%)	280 (50.1%)	269 (50.1%)
Survival			
Alive	239 (57.6%)	287 (51.3%)	445 (82.9%)

### 2.2 Construction and validation of a platelet-related risk score

We utilized the GSE136337 dataset acquired from the GEO dataset as a training cohort to create the risk score model associated with platelets. In order to determine PRGs with prognostic significance, we performed univariate Cox regression analysis on the genes under consideration. A significance threshold of *p* < 0.001 was applied to screen for genes with potential prognostic associations. Subsequently, a platelet-related risk model was constructed using Least absolute shrinkage and selection operator (LASSO) Cox regression. The model’s coefficients were derived from the previous step. By applying this model, a platelet-related risk score was calculated for MM patients in each dataset. In particular, the GSE4204 and GSE24080 datasets served as validation cohorts. In order to enhance patient stratification, the individuals in each dataset were divided into cohorts of high-risk and low-risk, depending on the risk score median specific to each dataset.

Survival disparities between different risk groups were compared by generating survival curves and dot plots. The R language package “stringr” was employed to create heat maps, enabling a comparison of platelet-related gene expressions across multiple datasets. Furthermore, receiver operating characteristic (ROC) curves were utilized to confirm the sensitivity and specificity of genes associated with platelets.

### 2.3 Comparative analysis of clinical characteristics

Univariable and multivariable Cox regression analyses were utilized to assess the influence of independent prognostic factors on the overall survival in both the training and validation cohorts. In addition, in the GSE136337 dataset, we examined clinical characteristics and compared risk scores between subgroups to identify potential subgroup differences.

### 2.4 Immune-related analysis and immune treatment sensitivity of the platelet-related model

To minimize variations arising from different algorithms, we employed multiple algorithms to evaluate the immune microenvironment of the subgroups. Specifically, estimating the proportion of immune and cancer cells (EPIC) (Racle et al., 2017), xCell (Aran et al., 2017), and single-sample genome enrichment analysis (ssGSEA) were utilized for this purpose. Furthermore, the correlations between eight platelet-related genes and immune cells were assessed using the cell-type identification by estimating relative subsets of RNA transcripts (CIBERSORT) method (Newman et al., 2015). Utilizing these methodologies, potential links between platelet-related genes and populations of immune cells can be explored. Additionally, the immune cell microenvironment scores of high-risk and low-risk groups were assessed and compared through the implementation of xCell and immunophenotype score (IPS) techniques (Charoentong et al., 2017). This allowed us to obtain knowledge about the variations in the composition of the immune microenvironment among these subgroups. Moreover, we assessed the differences in immune checkpoint responsiveness between the groups at high risk and low risk, enabling us to evaluate the possible consequences for the response to immunotherapy.

### 2.5 Drug sensitivity prediction

To compare the drug susceptibility between the low- and high-risk groups, the R package “pRRophetic” was utilized, enabling a comprehensive assessment of differences in drug response and sensitivity.

### 2.6 Validation of mutations and interaction network linked to platelet externally using online databases

To validate the cellular expression of PRGs, the Cancer Cell Line Encyclopedia database (CCLE) was utilized. The CCLE database can be browsed through this link: https://portals.broadinstitute.org/ccle. In order to examine the interactions between proteins (PPIs) associated with platelet-related genes, we obtained the PPI network linked to PRGs from the Search Tool for the Retrieval of Interaction Gene/Proteins (STRING) database (version 11.5) (https:// www.string-db.org/).This network analysis provided insights into the molecular interactions and potential functional relationships among these genes.

### 2.7 Gene set enrichment analysis

In order to explore potential underlying mechanisms linked to the platelet-related genes, we carried out pathway analysis utilizing the Kyoto Encyclopedia of Genes and Genomes (KEGG) pathways. We assessed enriched pathways across various datasets by utilizing the Gene Set Enrichment Analysis (GSEA) v4.0.2 software (http://software.broadinstitute.org/gsea/login.jsp). Our analysis considered statistical significance as *p* < 0.05 and q < 0.25.

### 2.8 Construction and validation a combined predictive nomogram

Utilizing the results obtained from univariable and multivariable Cox regression analyses, we created a combined nomogram facilitating prognostication of the overall survival rates at 1-year, 3-year, and 5-year for MM patients. This nomogram incorporated age, ISS stage, and the platelet-related risk score as prognostic factors. To validate the performance of the nomogram, calibration curves were plotted to assess its accuracy in predicting patient outcomes. In addition, we assessed the predictive abilities of ISS stage, platelet-related risk score, and the nomogram through the application of time-dependent ROC curves for the survival time points at 1 year, 3 years, and 5 years. Decision curve analysis (DCA) was performed to assess the clinical usefulness of every individual clinical characteristic and the risk score. This analysis allowed us to assess the net benefits of each factor in terms of survival prediction.

### 2.9 Cell lines and patients

The LP-1 and MM1.R cell lines, referred to as MM cell lines, were acquired from Fenghui Biotechnology Co., Ltd in Hunan, China. The cultivation of these cell lines took place in a controlled environment within a humid incubator set at 37°C with 5% CO_2_. The study included a total of 25 MM patients and 15 healthy individuals, and their clinical characteristics are presented in Supplementary Table S2. The First Affiliated Hospital of Wenzhou Medical University’s ethical committee granted the study permission. All study participants gave their informed consent, and the research followed the guidelines set forth in the Helsinki Declaration.

### 2.10 Quantitative real-time PCR

Bone marrow puncture was performed on 25 patients and 15 healthy people in the control group. And 5 mL of aspirate was taken from their bone marrow. Bone marrow mononuclear cells (BMMNC) were then isolated using density gradient centrifugation. We employed the Righton DNA&RNA Blood and Tissue Kit (supplied by Righton Bio, Shanghai, China) to perform total RNA isolation from the bone marrow of clinical MM patients and healthy volunteers, adhering to the guidelines provided by the manufacturer. To generate complementary DNA (cDNA), cDNA synthesis kits (obtained from Vazyme, Nanjing, China) were utilized in the subsequent reverse transcription step. To conduct PCR amplification, we employed the Taq Pro Universal SYBR qPCR Master Mix (Vazyme, Nanjing, China) in accordance with the guidelines provided by the manufacturer. For quantifying the expression levels of PRGs, we utilized quantitative reverse transcription PCR (qRT-PCR). To serve as an internal reference gene, β-ACTIN was selected, and the primers specified in Table 2 were applied. To ensure accuracy and reproducibility, each sample was subjected to three repetitions.

**TABLE 2:** Primers used in the study.

Gene symbol	Polarity	Sequence 5′–3′
CISH	forward	CTG​CTG​ATA​CCC​GAA​GCG​ACA
	reverse	GTT​GAT​GAC​AAG​GCG​GCA​CA
TAGLN2	forward	ACC​CAG​TGC​CGA​AAG​GAT​GT
	reverse	GAA​GAT​GTC​AGT​GGT​GTT​AAT​GCC
FLNA	forward	GGG​ACA​GAA​GGG​CAC​GGT​A
	reverse	CAG​GCA​CTC​GGG​TTA​CAG​G
KIF23	forward	GAA​GTG​GGA​GAA​AGA​ATG​TGA​GC
	reverse	CAG​TTT​TAG​GTT​CGG​TAA​CAA​TAG​C
H2BS1	forward	AGA​AGG​ACG​GCA​GGA​AGC​G
	reverse	TTGTAATGCGGCAGGCGG
IKBKG	forward	GAG​CAG​CGT​GGT​GGG​CAG​T
	reverse	CGG​AAC​GGT​CTC​CAT​CAC​AAT​C
PTEN	forward	AAG​ACC​ATA​ACC​CAC​CAC​AGC
	reverse	TCA​TTA​CAC​CAG​TTC​GTC​CCT
CTSW	forward	GAG​TTA​CCT​GAG​CCC​AGA​AGA
	reverse	GCCCTCCGATAGCCATAG
*β*-ACTIN	forward	TCA​AGA​TCA​TTG​CTC​CTC​CTG​AG
	reverse	ACA​TCT​GCT​GGA​AGG​TGG​ACA

### 2.11 Statistical analysis

For the purpose of these tasks, multiple software programs were utilized to conduct clinical evaluations and statistical analyses. The software programs employed included GraphPad Prism version 9.0.0 by GraphPad Software Inc. in San Diego, CA, United States, SPSS version 25.0 by SPSS Inc. in Chicago, IL, United States, as well as the widely used R software developed by the R Foundation for Statistical Computing in Vienna, Austria. In order to pinpoint the potential PRGs, we carried out univariate Cox regression analysis and LASSO regression analysis. Following that, we conducted multivariate Cox regression analysis to evaluate the predictive worth of the platelet-related risk score and clinical characteristics. We compared the survival rates through the utilization of Kaplan-Meier curves and log-rank test. To examine variables that followed a normal distribution, we utilized the independent *t*-test to make comparisons between groups. Categorical variables, on the other hand, underwent analysis using the Chi-square test. In situations where the distribution was non-normal, we employed the Mann-Whitney *U* test to compare two groups. For this particular investigation, we deemed a significance level of *p* < 0.05 as statistically meaningful. Regarding graphical representations, *p*-values were denoted as follows: *: *p* < 0.05; **: *p* < 0.01; ***: *p* < 0.001; ****: *p* < 0.0001. Additionally, the label “ns” conveyed a lack of statistical significance.

## 3 Results

### 3.1 Subject selection and baseline covariates

In this research, we conducted an examination on the survival information of 1511 individuals diagnosed with multiple myeloma. The data was collected from three datasets, specifically GSE136337, GSE4204, and GSE24080. The Cox regression analysis for uni- and multi-variables included subjects with relevant clinical co-variates from the training cohort (N = 415; GSE136337) and the validation cohort (N = 559; GSE24080). Unfortunately, due to insufficient clinical information, further Cox regression analyses could not be conducted on the second validation cohort (N = 537; GSE4204). Table 1 presents the clinical information for all three datasets, providing a comprehensive overview of the relevant patient characteristics.

### 3.2 Construction of a prognostic platelet-related risk score

**FIGURE 2:**
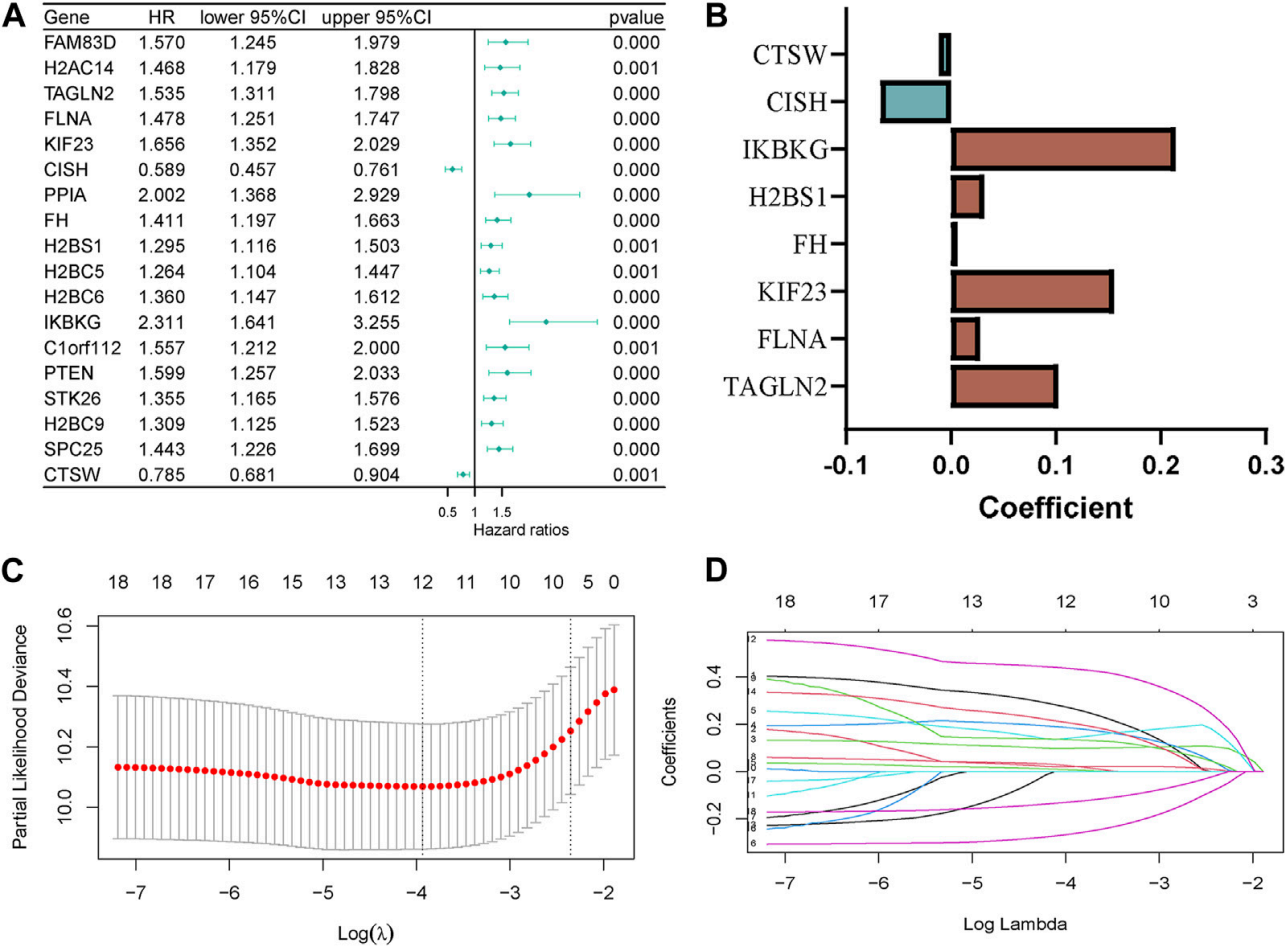
Construction of the platelet-related model. **(A)** Forest plot of hazard ratios manifesting the prognostic values of platelet-related genes. **(B)** LASSO coefficients of the 8 predictor genes for constructing the prognostic model. **(C,D)** LASSO Cox regression analysis for variable selection.

In the GSE136337 training cohort, we identified 18 platelet-related genes that showed significant associations with survival through univariable Cox regression analyses (*p* < 0.001). This is depicted in Figure 2A. To construct the platelet-related risk score, we applied LASSO Cox regression analysis and selected eight genes with high coefficients (Figures 2B–D). Among these genes, Transgelin 2 (TAGLN2), Filamin A (FLNA), Kinesin Family Member 23 (KIF23), Familial hypercholesterolemia (FH), H2B clustered histone 12 like (H2BS1, also known as H2BC12L), and Inhibitor of nuclear factor kappa B kinase regulatory subunit gamma (IKBKG) were identified as high-risk genes, while Chromogenic *in situ* hybridization (CISH) and Cathepsin W (CTSW) were labeled as low-risk genes. The following formula was used to determine the platelet-related risk score: platelet-related risk score = (0.1023 × expression of TAGLN2) + (0.0277 × expression of FLNA) + (0.1552 × expression of KIF23) + (0.0067 × expression of FH) + (0.0319 × expression of H2BS1) + (0.2144 × expression of IKBKG)—(0.0672 × expression of CISH)—(0.0115 × expression of CTSW). By employing this equation, platelet-related risk scores were computed for every individual in both the training and validation cohorts. Subsequently, employing the median value of each set, the specimens were categorized as high-risk or low-risk groups.

### 3.3 The prognostic capacity of the platelet-related risk score

**FIGURE 3:**
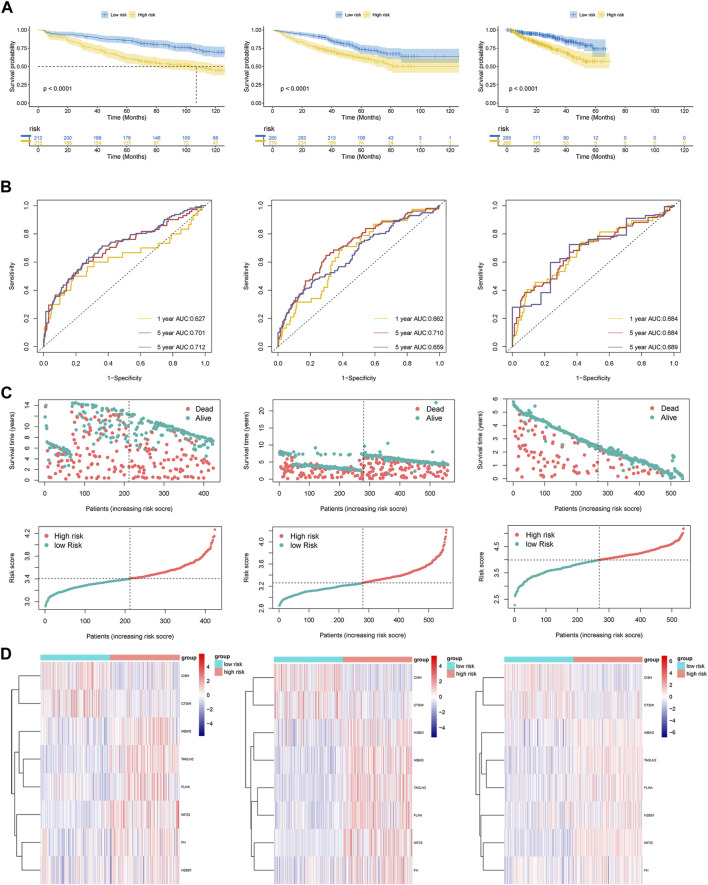
**(A)** Kaplan-Meier curves of patients in the high- and low-risk group (*p* < 0.0001). **(B)** The AUC of the model assessed by time-dependent ROC curves. **(C)** Dot plots comparing outcomes of subjects in the high- and low-risk cohorts. **(D)** The heat map displays results for the eight genes in both the training and validation cohorts.

Using Kaplan-Meier curves, we examined the differences in survival rates between the high-risk and low-risk cohorts. Our results revealed that patients categorized as high-risk exhibited inferior survival outcomes in comparison to those classified as low-risk across all datasets (Figure 3A). These findings were further supported by the dot plots (Figure 3C). In the dot plots, dead and alive points represented the survivors and deaths in each dataset respectively. And detailed data have been shown in Table 1. The overall survival time for alive and dead points were elevated in the low-risk group compared to the high-risk group, which showed consistent patterns of worse survival in the high-risk groups in each dataset. In order to assess the precision and accuracy of the platelet-related risk score, a time-dependent ROC analysis was performed. Within the GSE136337 training dataset, the AUC for survival at 1-year, 3-year, and 5-year intervals were found to be 0.627 (95% CI: 0.498–0.756), 0.701 (95% CI: 0.628–0.775), and 0.712 (95% CI: 0.658–0.776) correspondingly, as shown in Figure 3B. Furthermore, we compared the expression patterns of eight PRGs across the three datasets using heat maps. Consistent with the previously discussed formula for platelet-related gene expression, the high-risk group displayed lower expression levels of CISH and CTSW, while the other six genes exhibited an opposite trend (Figure 3D). Notably, the time-dependent ROC curves, heat maps, and dot plots of GSE24080 and GSE4204 demonstrated similar trends to those observed in GSE136337.

### 3.4 Comparative analysis of clinical characteristics and drug sensitivity

We utilized both univariate and multivariate Cox regression analyses to evaluate the forecasting potential of the platelet-related risk score. Additionally, the influence of various clinical factors such as sex, age, albumin, β2-microglobulin, lactic dehydrogenase (LDH), ISS, and R-ISS stage were examined utilizing identical methods in the GSE136337 and GSE24080 datasets (Table 3). In the training dataset, the multivariate analysis revealed a HR of 2.001 (95% CI: 1.467–2.730; *p* < 0.001) for the platelet-related risk score. Similarly, in the validation dataset, the multivariate analysis showed a HR of 1.530 (95% CI: 1.117–2.097; *p* = 0.008) for the platelet-related risk score. The findings of the study demonstrated that the platelet-related risk score maintained its independent association with survival outcomes.

**TABLE 3:** Univariate and multivariate Cox regression analyses of survival in the training and validation cohorts.

Characteristics	Training cohort GSE136337 (n = 415)				Validation cohort GSE24080 (n = 559)			
	Univariate analysis		Multivariate analysis		Univariate analysis		Multivariate analysis	
	Regression coefficient (SE)	*P*	Hazard ratio (95% CI)	*P*	Regression coefficient (SE)	*P*	Hazard ratio (95% CI)	*P*
Age (<65 vs. ≥65 years)	0.580 (0.156)	<0.001	1.754 (1.284–2.373)	<0.001	0.198 (0.178)	0.267	-	-
Sex (female vs. male)	−0.246 (0.154)	0.11	-	-	−0.03 (0.155)	0.848	-	-
Albumin (≥3.5 vs. <3.5 g/dL)	0.409 (0.177)	0.021	-	-	0.653 (0.190)	0.001	-	-
β2m (<3.5 vs. 3.5–5.5 vs. ≥5.5 mg/L)	0.424 (0.091)	<0.001	-	-	0.544 (0.088)	<0.001	-	-
LDH (≤250 vs. >250 U/L)	0.729 (0.270)	0.007	-	-	1.347 (0.195)	<0.001	-	-
del (17p)	0.009 (0.417)	0.812	-	-	-	-	-	-
t (4,14)	0.036 (0.455)	0.936	-	-	-	-	-	-
t (14,16)	0.721 (1.004)	0.472	-	-	-	-	-	-
ISS (Ⅰ vs. Ⅱ vs. Ⅲ)	0.502 (0.095)	<0.001	1.519 (1.108–2.083)	0.009	0.558 (0.091)	<0.001	1.644 (1.369–1.974)	<0.001
R−ISS (Ⅰ vs. Ⅱ vs. Ⅲ)	0.594 (0.133)	<0.001	1.043 (0.656–1.659)	0.857	-	-	-	-
Risk (low vs. high)	0.783 (0.157)	<0.001	2.001 (1.467–2.730)	<0.001	0.613 (0.165)	<0.001	1.530 (1.117–2.097)	0.008

Albumin, β2M, and LDH, were not included in the multivariate analysis, because of co-linearity with the ISS, or R-ISS.

**FIGURE 4:**
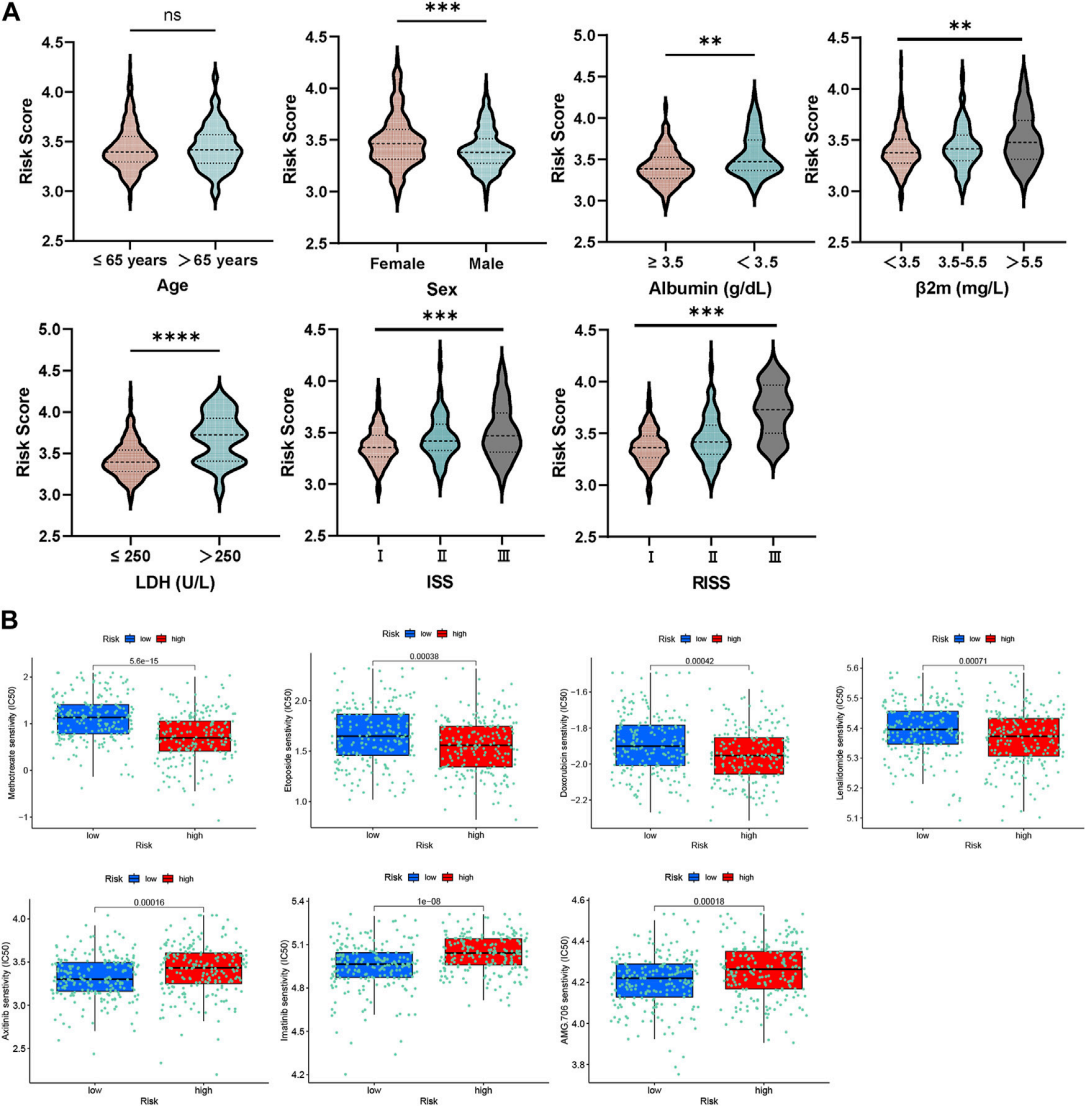
**(A)** Relationship between risk score and distinct clinical traits. **(B)** Evaluations of the drug susceptibility among the subtypes.

Our study conducted an investigation into the correlation between risk scores and various clinical features in GSE136337. Notably, higher levels of LDH, albumin, and β2-microglobulin were consistently observed to be positively correlated with higher risk scores, as illustrated in Figure 4A. Additionally, the study observed a progressive increase in risk scores with higher ISS or R-ISS staging, suggesting a direct relationship between disease severity and the platelet-related risk score.

Remarkably, this study examined the responsiveness of the group at high-risk in comparison to the low-risk group towards different therapeutic agents. The outcomes indicated that the high-risk participants displayed a resistant response to etoposide, methotrexate, lenalidomide, and doxorubicin, as affirmed by the decreased half maximal inhibitory concentration (IC50) values recorded for these drugs (Figure 4B). On the other hand, the group at high-risk exhibited increased sensitivity to AMG.706 (motesanib), axitinib and imatinib, which are vascular endothelial growth factor receptor inhibitors.

These findings provide valuable insights into the relationship between platelet-related risk score and clinical features, further highlighting the potential implications for personalized treatment strategies based on risk stratification.

### 3.5 Immune-related analysis and immune treatment sensitivity using platelet-related risk score

The group comparison charts, which were created using different algorithms, highlighted the disparities in the immunological microenvironment between the high-risk and low-risk groups.

**FIGURE 5:**
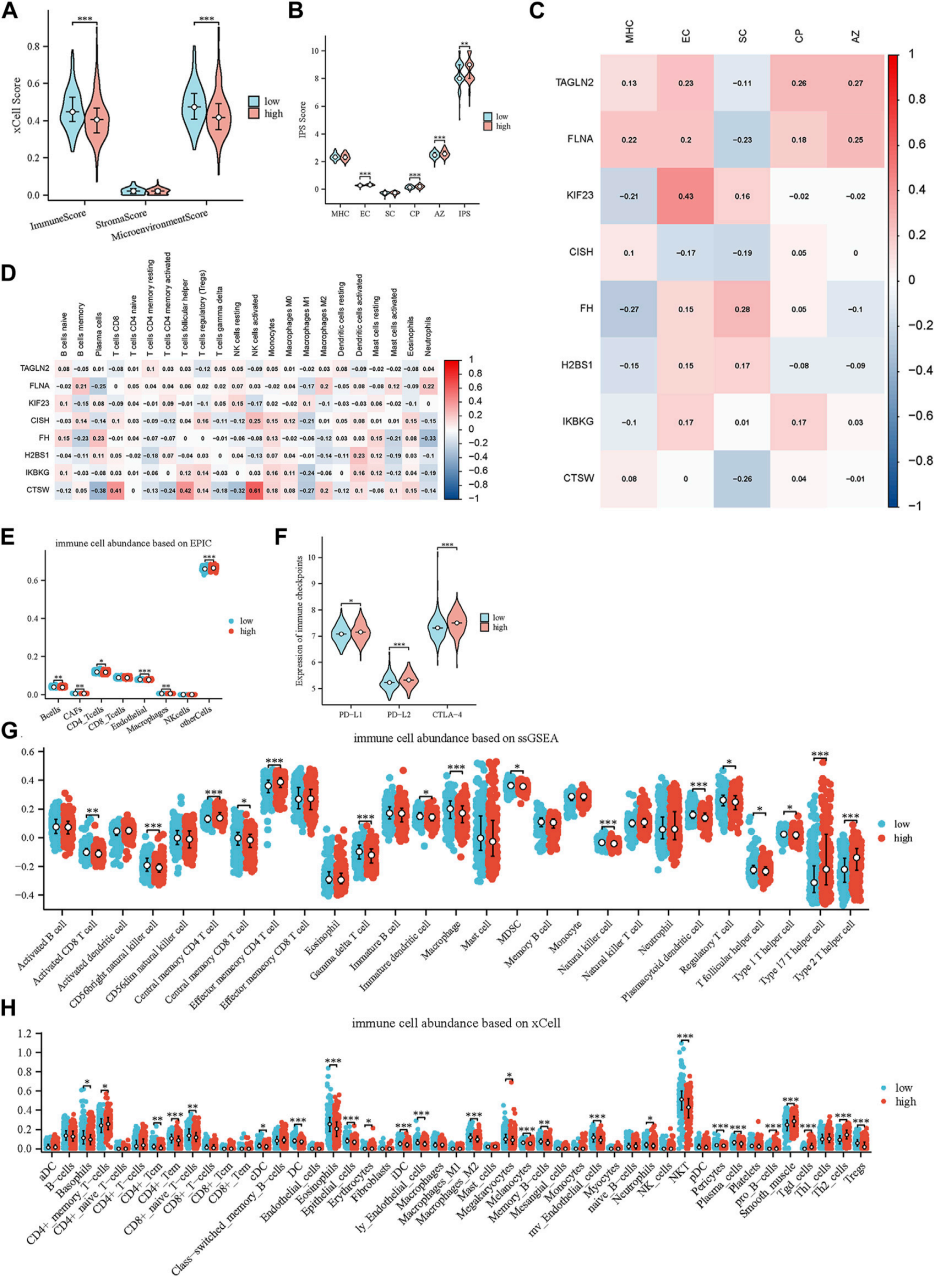
Characterization of the tumor microenvironment and immune treatment sensitivity of the glycolytic model. **(A)** Immune-related scores calculated by xCell. **(B)** Immune-related scores calculated by IPS. **(C)** Relationships of prognostic genes with distinct immune phenotypes. **(D)** Relationships of prognostic genes with immune cells. **(E)** Comparison of immune cell infiltration levels between high- and low-scoring cohorts using EPIC. **(F)** Comparison of 3 immune checkpoints expression between high- and low-risk groups. **(G, H)** Comparison of immune cell infiltration levels between high- and low-scoring cohorts using ssGSEA and xCell.

The low-risk group outperformed the high-risk group in terms of immunological and microenvironment scores, as determined by the xCell technique. The group at high risk exhibited a slightly elevated stromal score while it was not statistically significant. These results imply an increased level of immune cell infiltration in the group at low risk, as well as a greater level of stromal cell infiltration in the high-risk group (Figure 5A).

Furthermore, we compared the differences in immune cells and stromal cells between the high-risk and low-risk groups using the Epic, xCell, and ssGSEA methods (Figures 5E,G,H). Across all three approaches, we consistently noticed an increased extent of immune cell infiltration within the low-risk group. This encompassed the presence of γδ T cells, NK cells, effector CD4^+^ T cells and activated B cells. Intriguingly, we also detected favorable associations between CISH and CTSW expression and distinct immune cell subtypes, including activated NK cells, activated CD4^+^ memory T cells and CD8^+^ T cells, as determined by the application of the CIBERSORT technique (Figure 5D). These cells of the immune system are essential players in the process of combating tumors and are linked to a positive prognosis for patients. These findings further support and validate our initial hypothesis.

We also explored the variance in immune checkpoint molecule expression among the high-risk and low-risk clusters. Remarkably, our findings revealed that the high-risk cohort demonstrated elevated levels of immune checkpoint molecule expression, encompassing PD-L1, PD-L2, and CTLA-4. (Figure 5F). These molecules play an essential function in governing the immune response against tumors and inhibiting the activity of immune cells (Yi et al., 2021; Fan et al., 2022). The potential justification for targeted immunotherapeutic interventions is indicated by the observed increase in PD-L1, PD-L2, and CTLA-4 among individuals in the high-risk group.

Moreover, we utilized the IPS to further assess and compare the immune-related scores between the high-risk and low-risk groups. The IPS incorporates four distinct immune phenotypes: antigen presentation (AP), effector cells (EC), suppressor cells (SC), and checkpoints (CP). The comprehensive measure of tumor immunogenicity, known as the IPS z-score, incorporates the aforementioned scores and enables the prediction of immune checkpoint inhibitor (ICI) therapy response in different cancer types (Givechian et al., 2018). When comparing the groups at high risk and low risk, it was observed that the high-risk group exhibited higher scores for EC, CP, and AZ (Figure 5B). Based on this discovery, it is implied that individuals in the high-risk category might demonstrate heightened receptiveness to ICI therapy. This aligns with the prior analyses conducted on the disparities in immune checkpoint function between these two cohorts. Furthermore, we examined the correlation of IPS scores with eight genes, and we observed a negative correlation between the protective genes (CISH and CTSW) and immunosuppressive cells, as well as a positive correlation with antigen presentation (Figure 5C). These results align with our previous findings, further supporting the notion that platelet-related genes may serve as valuable indicators of the immune status in MM patients.

### 3.6 Investigation of biology functions based on platelet-related risk score

**FIGURE 6:**
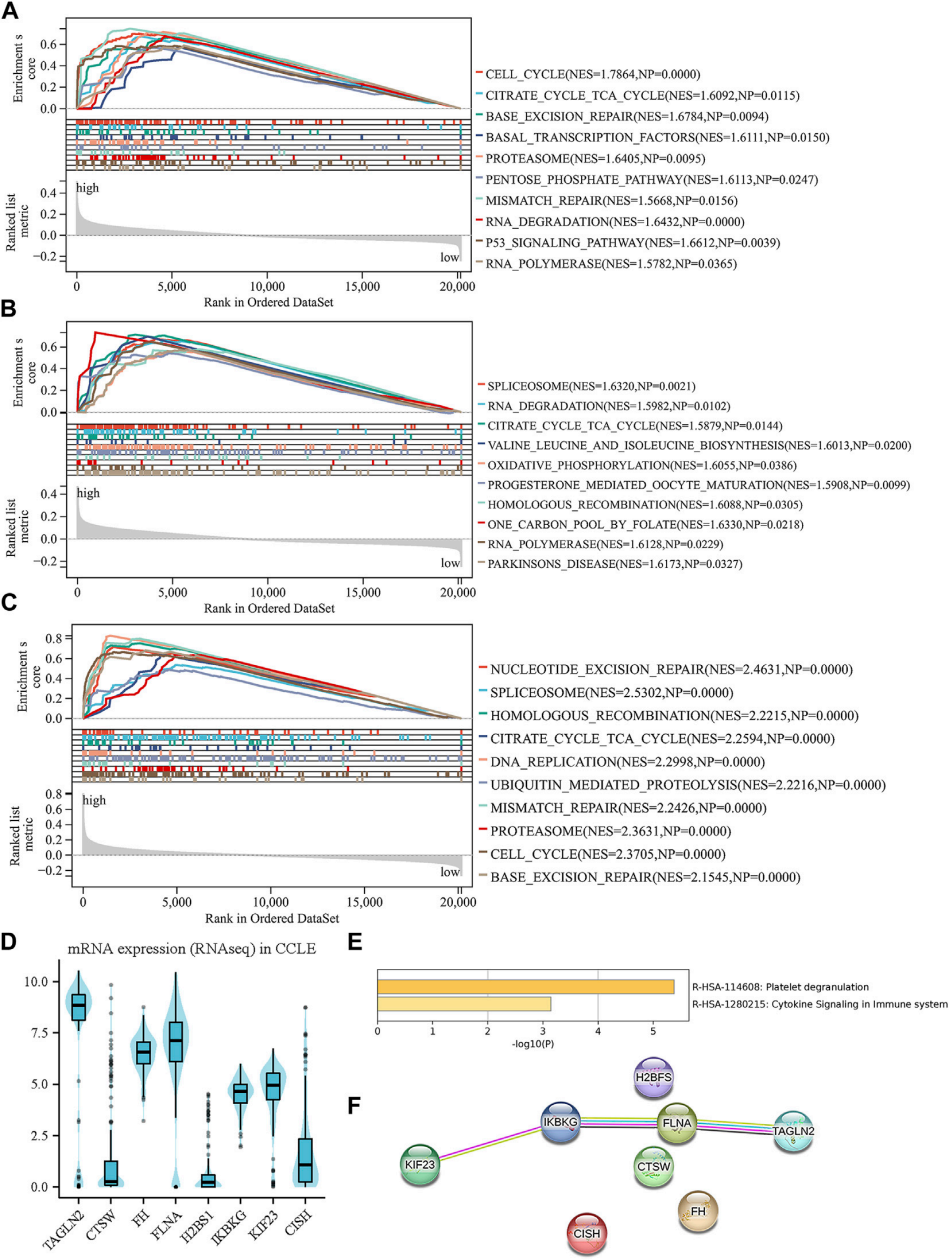
Investigation of biology functions based on prognostic platelet-related signature. **(A–C)** enriched pathways of the high-risk group in each cohort. **(D)** The external validation of the expression levels of the eight genes using CCLE. **(E)** Enriched pathways among the eight PRGs obtained from Metascape. **(F)** Protein–protein interactions among the eight PRGs obtained from STRING.

A thorough examination was carried out to investigate the biological functions linked to both the high-risk and low-risk groups. To delve into the enrichment of KEGG pathways associated with genes related to platelets, GSEA was conducted on each dataset (Figures 6A–C). By performing this analysis, a notable clustering of enriched pathways was observed in the high-risk group, such as proteasome pathway, cell cycle, DNA mending, nucleotide elimination mending, along with the one-carbon reservoir of folate, which display close associations with the progression of tumors.

After examining the Matascape database, we discovered two pathways that exhibited a higher abundance of eight genes related to platelets (Figure 6E). These pathways were cytokine signaling in the immune system and platelet degranulation. Furthermore, using the STRING database, we conducted an analysis of the interactions that occur between the eight PRGs. The results revealed associations between TAGLN2, KIF23, FLNA, and IKBKG, indicating potential interactions and functional relationships between these genes (Figure 6F). The biological mechanisms underlying the high-risk group and the role of platelets in the progression of MM are illuminated by these discoveries. Our understanding of the complex interplay between platelets and tumor development in MM is enhanced by the pathways and protein-protein interactions that have been identified.

### 3.7 External validation using online databases

The CCLE database revealed that TAGLN2, FH, FLNA, IKBKG, and KIF23 were significantly upregulated at the cellular level. In contrast, CISH and CTSW displayed downregulation in the cellular level. It is worth noting that the expression patterns of these genes except for H2BS1 align with the previously mentioned model formula, indicating their consistency with the platelet-related risk score (Figure 6D). These results offer more proofs for the biological significance and applicability of the discovered platelet-related genes in the setting of multiple myeloma.

### 3.8 Construction and validation of the combined nomogram

**FIGURE 7:**
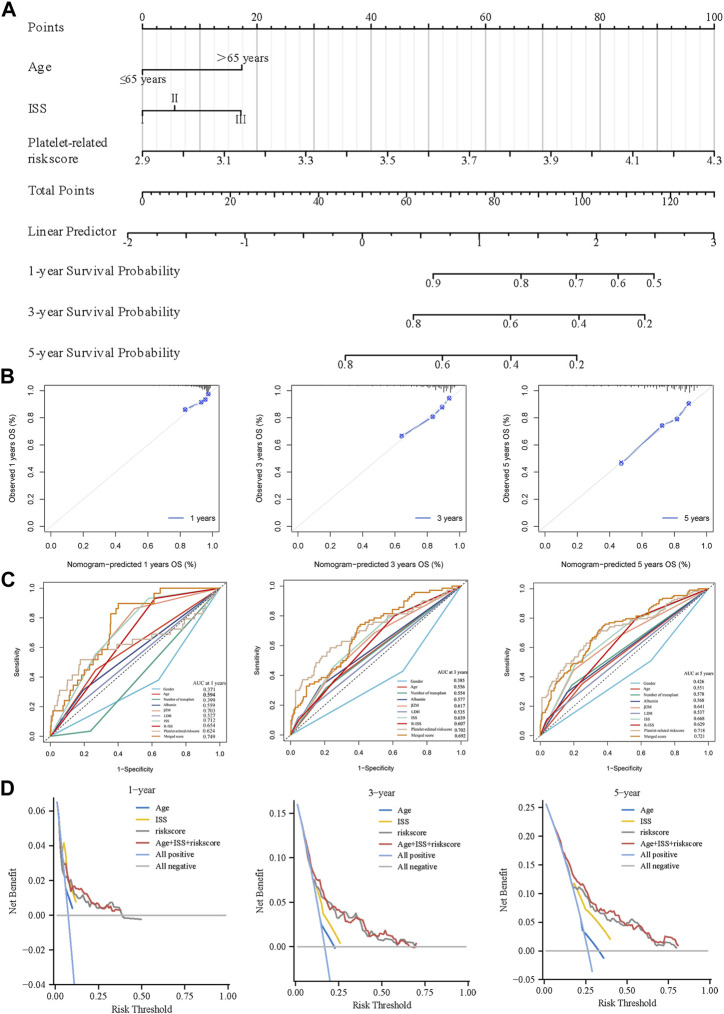
Creation of a predictive nomogram to evaluate clinical application. **(A)** The nomogram for the training cohort based on age, ISS phase, and glycolytic risk score. **(B)** To verify the accuracy of the 1-, 3-, and 5-year survival predictions, calibration plots were created. **(C)** Combined with various clinical co-variates and time-dependent ROC analyses at 1, 3, and 5 years. **(D)** DCA were used to determine the survival net benefits of each clinical trait and the risk score.

Age and platelet-related risk scores were integrated into combined nomograms to predict 1-, 3-, and 5-year survival rates (Figure 7A). Calibration plots were employed to evaluate the performance of the nomograms in forecasting survival rates over the specified timeframes. Notably, the calibration plots of the training cohort demonstrated a high degree of consistency between the predicted and actual values of 1-, 3-, and 5-year (Figure 7B). By incorporating age, ISS, and the platelet-related risk score, the nomogram significantly enhanced the accuracy of 1-, 3-, and 5-year survival predictions in the training cohort. The AUC of 1-, 3-, and 5-year improved from 0.717, 0.639, and 0.668 (using ISS alone) to 0.749, 0.692, and 0.721 (using the nomogram) respectively, indicating the superior performance of the nomogram in survival prediction (Figure 7C). Similarly, the validation dataset (GSE24080) also demonstrated improved prediction accuracy with the nomogram, yielding AUC values of 0.718, 0.687, and 0.692 for 1-, 3-, and 5-year survival respectively, compared to AUC values of 0.677, 0.634, and 0.647 obtained using ISS alone. The performance of the nomogram surpassed other metrics when assessed using DCA curves. Notably, the platelet-related risk score demonstrated superior net gain in survival at 1, 3, and 5 years, further reinforcing its role as a valuable independent prognostic marker (Figure 7D). Using the same method, the nomogram was validated at GSE24080, and the related images are shown in Supplementary Figure S1. In summary, the platelet-related risk score serves as an additional and reliable prognostic marker, complementing the conventional ISS stage.

### 3.9 External validation using qRT-PCR

**FIGURE 8:**
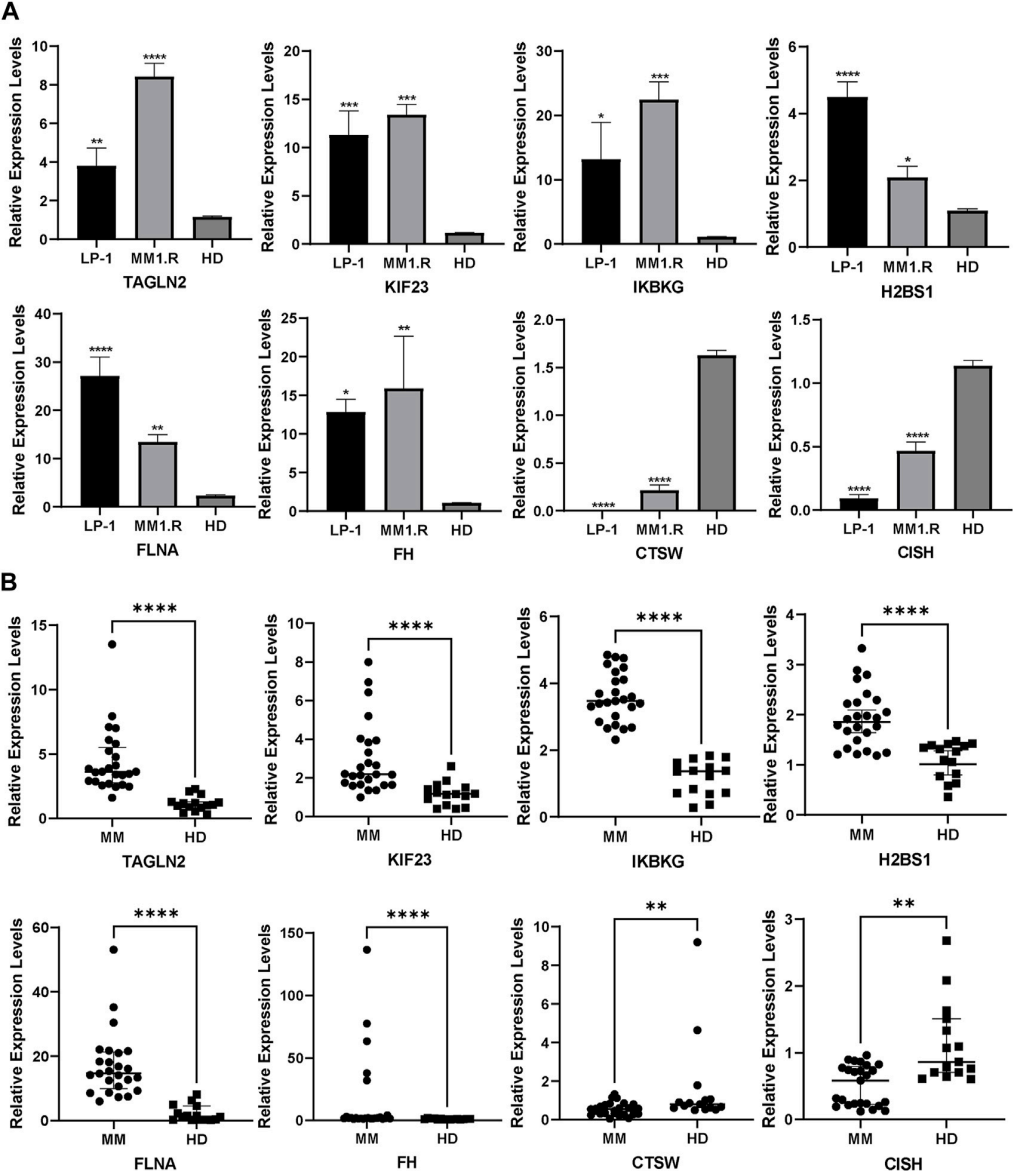
External validation in MM cell lines **(A)** and patients **(B)** using qRT-PCR (Mean ± SEM). ns, no significance; *p < 0.05; **p < 0.01; ***p < 0.001; ****p < 0.0001.

To further assess the predictive capacity of our identified platelet-related genes in MM, we performed qRT-PCR experiments on MM cell lines and patient samples. In line with the platelet-related risk score formula, the expression levels of TAGLN2, FLNA, KIF23, FH, H2BS1 and IKBKG displayed an upregulation trend in MM1.R and LP-1 MM cell lines (Figure 8A). Conversely, CTSW and CISH exhibited a downregulation trend in these cell lines, consistent with the predicted pattern based on the formula.

Moreover, we extended our analysis to MM patient samples, comparing the expression of the PRGs with normal controls. The results demonstrated that CISH and CTSW expression was lower in MM patient samples compared to the normal controls, while the other six genes exhibited an opposite trend, showing higher expression levels in MM patient samples (Figure 8B).

## 4 Discussion

In recent years, novel antitumor agents such as proteasome inhibitors and immunomodulatory drugs have led to advances in overall survival (OS) in multiple myeloma (Rossi et al., 2013). However, most patients will experience multiple relapses. Eventually, the remissions become shorter in duration, and the patients die from treatment-related complications or the disease itself (van de Donk et al., 2021). The complexity and instability of the genome in multiple myeloma significantly impact treatment outcomes (Keats et al., 2012; Miller et al., 2017). To increase these patients’ chances of survival, a more precise OS prediction model for targeted therapy or immunotherapy is therefore required.

The role played by platelets in multiple myeloma is complex. In previous studies, P-selectin was demonstrated as a cellular adhesion molecule that interacted with activated endothelial cells positioned on the blood vessels’ surface and activated platelets (Daniëlle M et al., 2017). Studies have shown that P-selectin plays a crucial role in the initial metastatic phases of cancer through its interaction with circulating malignant cells (Hans-Åke et al., 2021). Furthermore, scientific experiments have provided evidence of a substantial rise in P-selectin levels among individuals with recently diagnosed MM when compared to those who are in good health (Dorota et al., 2013). This occurrence could potentially stem from the activation of platelets and heightened infiltration of myeloma cells (Abdel Kareem et al., 2011). Furthermore, platelets release soluble factors such platelet factor 4, CD40 ligand, and P-selectin, which contribute to cancer-associated thrombosis (Hans-Åke et al., 2021). And it has been shown that people with cancer exhibit these indicators of platelet activation at higher levels (Hans-Åke et al., 2021). For example, cancer cells have been shown to produce CD40L, which stimulates platelet aggregation and activation. And in this way promotes tumor metastasis (Wiktoria et al., 2022). Due to these different mechanisms, platelets may represent potential therapeutic targets.

Therefore, we created a prognostic model that was defined by eight genes connected to platelets based on the dataset of GSE136337 in our research. Additionally, The model performed well on the external validation dataset GSE24080 and GSE4204. And we verified that this platelet-related gene profile was an independent factor of survival prognosis using multifactorial Cox regression.

As the protective prognosis marker, CISH plays a crucial role in the control of immune-related signaling pathways, impacting the polarization of lymphocytes and activation of myeloid cells through the regulation of downstream signaling molecules involved in key cytokines like IL-4, IL-6, and IFN-γ. Disruptions in CISH activity can disturb these cellular processes, resulting in the onset of inflammatory, autoimmune disorders and cancerous conditions (Sobah et al., 2021). CTSW is a member of the cysteine protease family. Most histones are found in antigen-presenting cells and are involved in antigen processing. It has been shown that CTSW is highly expressed in peripheral derived regulatory T cells (pTreg) cells and plays an important role in inhibiting pTreg cell differentiation and function (Li et al., 2023). And numerous experiments have shown that Treg cells not only inhibit anti-tumor immune responses, but also promote tumor microenvironmental vascular regeneration (Chaudary and Hill, 2007; Kim et al., 2020; Li et al., 2024). Thus CTSW may inhibit tumor development by suppressing Treg cell proliferation, which requires further experimental demonstration.

Many of the identified platelet-related genes have been found to have the potential to predict the prognosis of tumors and were related to the biological process of platelets. TAGLN2, FLNA, KIF23, H2BS1, IKBKG and FH were identified as negative prognostic factors. Among them, FLNA is linked to numerous biological processes, including cell signaling and motility. And it plays a crucial role in cell migration and adhesion, which puts it in close proximity to cancer invasion and metastasis (Kim et al., 2020). Recently, it has been shown that FLNA interacts with its platelet partners, including aIIbb3, the signaling pathway of the vascular hemophilia factor receptor GPIb-IX-V, tyrosine kinase, and collagen receptor glycoprotein VI (GPVI). And it is involved in platelet activation (Rosa et al., 2019; De Silva et al., 2022; Ellis et al., 2024). Moreover, TAGLN2 governs the dynamics of the cytoskeletal protein actin by reinforcing actin filaments and plays a crucial role in the remodeling procedures of the actin cytoskeleton, encompassing cellular proliferation, differentiation, migration, and programmed cell death (Dutta et al., 2021). Various studies have shown that potentially carcinogenic factor TAGLN2 is altered at the transcriptional and translational levels in a variety of malignancies, including as leukemia, breast cancer, colorectal cancer and lymphoma (Zhao et al., 2021; Pan et al., 2023). It has also been suggested that TAGLN2 is associated with angiogenesis (Zhao et al., 2021). However, the signaling pathways associated with it in relation to cancer development are currently unclear and need to be further investigated. KIF23 plays a key role in cell proliferation, metabolism, differentiation, metastasis and survival through the activation of PI3K/AKT/mTOR and Wnt/β/Linker protein signaling pathway (Huang et al., 2022; Jin et al., 2022), and has been demonstrated in cancers such as gastric cancer and diffuse large B-cell lymphoma (Gong et al., 2022; Bai and Liu, 2023). The role of other negative genes in tumors still needs to be further discussed and explored.

By biological function analysis, some pathways related to platelets and tumors were enriched in the high-risk group. Among them, the proteasome pathway, which is known to interact with platelets, was shown to be strongly related with the high risk group (Colberg et al., 2020). Furthermore, cellular pathways like cell cycle, DNA mending, nucleotide elimination mending, along with the one-carbon reservoir of folate, which display a close association with the progression of tumors, were also magnified in the group at high risk (Marteijn et al., 2014; Hopkins et al., 2022; Liu et al., 2022). In Matascape database, two pathways associated with platelet were found highly linked to the eight genes. These pathways were cytokine signaling in the immune system and platelet degranulation. Platelets play a pivotal role in the advancement of tumors through initiating and clumping onto the surface of tumor cells. This process prompts degranulation and subsequent shielding of tumor cells against identification and eradication by immune cells of the host (Wang et al., 2022; Liao et al., 2023). This phenomenon promotes accelerated tumor cell growth and facilitates metastasis. The enrichment of cytokine signaling in the immune system further supports the involvement of platelets in immune cell activities during the progression of multiple myeloma (Li et al., 2022), consistent with previous findings.

Additionally, we found positive correlations between the expression of positive genes and many immune cell subtypes, such as active CD8^+^ T cells, activated CD4^+^ memory T cells and activated NK cells. Within the tumor microenvironment (TME), effector CD4^+^ T cells and γδ T cells exert a crucial function in immune surveillance against tumor growth (Park and Lee, 2021). Throughout the progression of MM, the functionality of NK cells may undergo substantial modifications, thereby ultimately impeding the advancement of the disease (Pazina et al., 2021). Moreover, the activity of NK cells demonstrated a positive correlation with the duration of disease remission among patients diagnosed with MM (Pazina et al., 2021). Numerous investigations have demonstrated that malignant plasma cells enhance their own survival and proliferation by modulating the bone marrow microenvironment, and that immunosuppression thereby raises the risk of infection and subsequent cancer (Caro et al., 2022). The development of relapse and medication resistance in plasma cells, as well as their survival and proliferation, are all influenced by interactions within the bone marrow microenvironment in multiple myeloma patients (Yang and Lin, 2015). This is consistent with our findings. Furthermore, the high-risk groups exhibited increased immunogenicity and immune checkpoint expression levels. All of these findings point to a complicated mechanism that the platelet-related high-risk group may have that helps define the immune milieu and forecast immune-targeted therapy.

We discovered that there were differences in the pharmacological sensitivity between the low-risk and high-risk groups. The high-risk group that showed resistance to etoposide, methotrexate, lenalidomide and doxorubicin. All multiple myeloma patients get standard first-line therapy consisting of dexamethasone, an oral immunomodulatory drug (such as lenalidomide) and an injectable proteasome inhibitor (such as bortezomib) (Cowan et al., 2022). Lenalidomide as an immunomodulatory agent improves survival of multiple myeloma patients through anti-proliferative and immunomodulatory effects (McCaughan et al., 2022). In addition, The VDT-PACE chemotherapy regimen, which consists of bortezomib, dexamethasone, thalidomide, cisplatin, doxorubicin, cyclophosphamide and etoposide, is very beneficial for patients who have been diagnosed with severe diseases such plasma cell leukemia or extramedullary plasmacytomas (Kapoor et al., 2012). This shows that individuals with high platelet risk scores are resistant to typical treatment for multiple myeloma, implying a shorter survival time, which is consistent with our findings.

However, we found high sensitivity to vascular endothelial growth factor receptor inhibitors such as motesanib, imatinib, and axitinib in the high-risk group. This was consistent with our finding of high platelet risk scores. Previous studies have found axitinib in combination with chemotherapeutic or targeted agents improves antitumor efficacy in many tumor models such as non-small-cell lung cancer and renal cancer when compared to single agent treatment (Hoh et al., 2014; Bondarenko et al., 2015). Because the combinations are linked to the blockade of vascular permeability, angiogenesis, and concurrent induction of apoptosis in tumor cells (Liu et al., 2019). Motesanib and imatinib, as comparable inhibitors, have similar effects to axitinib (Liu et al., 2019). This is consistent with the strong ICI treatment response observed in the high-risk group mentioned earlier. And this may suggests vascular endothelial growth factor receptor inhibitors and immune checkpoint inhibitors may be new treatment options for multiple myeloma.

However, our study has some limitations. Firstly, GEO database is lack of specific treatment information so that we could not take treatment into account in the survival prognosis of patients. Secondly, our study is a retrospective analysis. So it is better to perform a prospective multicenter study. Thirdly, the platelet-related risk score included eight genes associated with survival, but contribution of each gene in this formula should be studied. The link between these genes and platelet-related biological processes and multiple myeloma remains unproven, and detailed mechanisms still need to be explored at the cellular and molecular levels. Ultimately, in terms of therapeutic analysis, further studies are needed to confirm the therapeutic benefits of antiplatelet drugs in MM.

In conclusion, our study firstly developed and validated a platelet-related risk score for MM patients, which was recognized as an independent factor influencing survival. The nomogram and created eight platelet-related gene signature demonstrated outstanding results in both internal and external cohorts. And the trend of platelet-related genes in MM patients was demonstrated in in vitro experiments. Drug selection for chemotherapy, targeted therapy and immunotherapy drugs can be more effectively directed for high-risk patients with the use of the platelet-related risk score and nomogram. In addition to indicating the prognosis in MM patients, platelet-related genes may also offer novel avenues for therapeutic research in multiple myeloma.

## Data Availability

The original contributions presented in the study are included in the article/Supplementary Material, further inquiries can be directed to the corresponding authors.

## Supplementary material

SUPPLEMENTARY FIGURE S1
**(A)** Combined with various clinical co-variates and time-dependent ROC analyses at 1, 3, and 5 years. **(B)** To verify the accuracy of the 1-, 3-, and 5-year survival predictions, calibration plots were created. **(C)** DCA curves were used to determine the survival net benefits of each clinical trait and the risk score.
