# Supplementary figures and images for: Identification and validation of a platelet-related signature for predicting survival and drug sensitivity in multiple myeloma
Source: Front Pharmacol. 2024 May 16;15:1377370. doi: 10.3389/fphar.2024.1377370 (PMC11137312; doi:10.3389/fphar.2024.1377370)

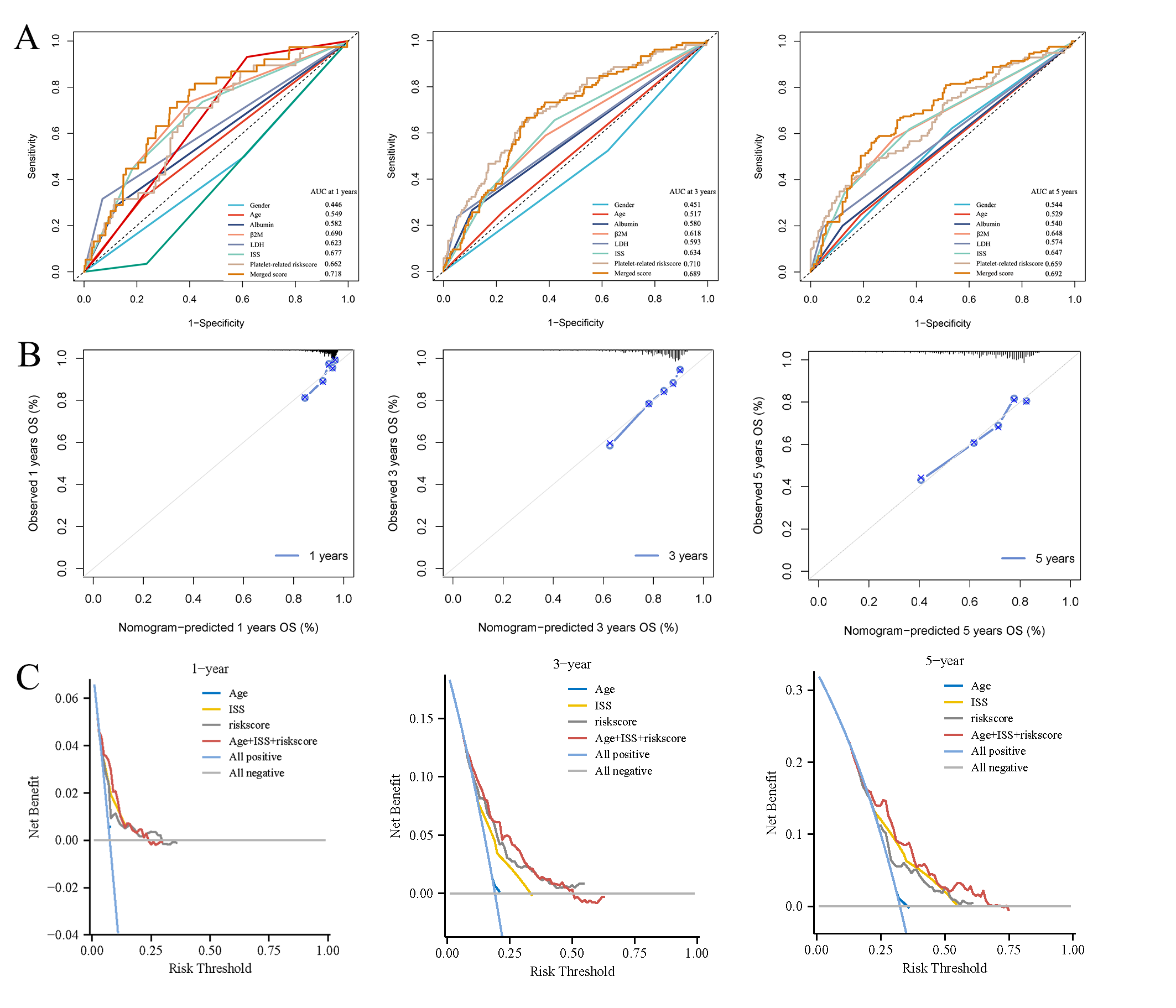

Supplement: Supplementary file 2 [file Image1.TIF]
